# Silencing of lncRNA AFAP1-AS1 suppressed lung cancer development by regulatory mechanism in *cis* and *trans*

**DOI:** 10.18632/oncotarget.20549

**Published:** 2017-08-24

**Authors:** Baoying Peng, Anfei Liu, Xuanwei Yu, Enwu Xu, Jiabin Dai, Mengcheng Li, Qiaoyuan Yang

**Affiliations:** ^1^ The State Key Lab of Respiratory Disease, The First Affiliated Hospital of Guangzhou Medical University, Yuexiu District, Guangzhou 510120, PR China; ^2^ The Institute for Chemical Carcinogenesis, Guangzhou Medical University, Xinzao, Panyu District, Guangzhou 511436, China; ^3^ Department of Thoracic Surgery, General Hospital of Guangzhou Military Command of Chinese People’s Liberation Army, Guangzhou 510010, China

**Keywords:** long noncoding RNA, AFAP1-AS1, lung cancer, natural antisense transcripts, AFAP1

## Abstract

Although the long noncoding RNA AFAP1-AS1 has been shown to be involved in various types of cancer, its involvement in lung cancer remains poorly understood. In the current study, we found that AFAP1-AS1 was substantially over expressed in lung cancer tissues and cell lines. In addition, AFAP1-AS1 expression level was proven to be associated with the malignant features of lung cancer. Knockdown of AFAP1-AS1 significantly suppressed cell proliferation by increasing cell apoptosis and G0/G1 phase retardation of cell cycle in lung cancer cells. Furthermore, AFAP1-AS1 knockdown could suppress tumor growth of lung cancer in BALB/c nude mice. We also identified that AFAP1-AS1 silencing could influence the expression of AFAP1 and KRT1 on mRNA and protein level by *cis* and *trans* regulatory mechanism. Moreover, the oncogenic activities of AFAP1-AS1 on cell proliferation are partially mediated by KRT1. In summary, these findings demonstrate that AFAP1-AS1 plays an essential role in promoting lung cancer development in *vitro* and *vivo*. It indicated that AFAP1-AS1 is a promising prognostic predictor for patients with lung cancer.

## INTRODUCTION

Lung cancer is one of the most common forms of cancer and leading cause of cancer deaths in males, and the fourth most commonly diagnosed and second leading cause of cancer deaths in females. It continues to be a major public health problem throughout the world. The incidence of lung cancer has dramatically increased in ten years, with more than 1.6 million new patients diagnosed each year with this disease [1, 2]. In spite of the progress in understanding the molecular mechanisms, and numerous medical and surgical treatments for lung cancer in recent years, the overall survival time of lung cancer patients has not changed greatly. With only 10-15 % of 5-year overall survival, lung cancer accounts for more than one quarter of all cancer deaths [3]. The most important factor for survival of lung cancer patients is the stage of the disease at diagnosis. Earlier detection and diagnosis of lung cancer could potentially lead to increase the number of treatment options available and thereby improving prognosis. Therefore, understanding the mechanisms of lung cancer progression and identifying effective biomarkers for diagnosis of lung cancer at early stage are vital for improving the therapy and overall prognosis of this disease.

Long noncoding RNAs (lncRNAs) are emerging as important regulators of disease processes including cancer. The recognition and identification of functional roles for lncRNAs has provided a new dimension to our understanding of cancer pathogenesis [4]. LncRNAs are a class of noncoding RNA over 200 nucleotides with no protein-coding potential [5]. Altered expression of lncRNAs has been reported in many types of cancer and in other disease settings [6-9]. Based on the emerging literature, lncRNAs may be useful in elucidating cancer pathogenesis and pathophysiology. Importantly, the ability of lncRNAs to alter basic cellular physiology by altering gene expression raises the possibility that aberrant expressions of lncRNAs may potentially contribute to cancer pathophysiology and tumorigenesis. Examples include HOTAIR in breast cancer and HCC [7, 8], MALAT1 in lung cancer and bladder cancer [9, 10], and many of these transcripts appear to act near their site of synthesis to regulate the expression of gene *in cis* or act *in trans*, indicating that lncRNAs may have important roles as therapeutic targets or as biomarkers of cancer [11-13].

Antisense transcript lncRNAs (AS lncRNAs) are a type of lncRNA molecules transcribed from the opposite DNA strand of protein-coding and non-protein-coding gene compared with sense transcripts and overlap in part with sense RNA [14]. Global transcriptomic analyses have confirmed that there are a number of natural antisense transcripts (NATs), which are recently recognized as important modulators for gene regulation [15]. It is considered that up to 70% of murine genomic loci existed NATs, most of which are lncRNA [16]. NATs appear to function in a highly cell type-specific manner, exerting in *cis* (affecting genes on the same chromosome from which they are transcribed) or in *trans* (affecting genes on another chromosome) effects on other genes involved in transcriptional and post-transcriptional regulation [17-19]. Compared with the stabilities of intergenic and intronic lncRNAs, AS lncRNAs are more stable [20]. The conservation and stability of AS lncRNAs might be good indicators for their potential biological functions.

Recently, a growing number of AS lncRNAs have been investigated and performed the regulation of their sense mRNA expression [21-23]. AFAP1-AS1 which located on the opposite strand of coding gene AFAP1, is a recently identified AS lncRNA. Up to now, several studies have proved that AFAP1-AS1 has been implicated tumorigenesis of various cancers. Increased expression of AFAP1-AS1 was found in Barrett esophagus, esophageal adenocarcinoma, pancreatic ductal adenocarcinoma, nasopharyngeal carcinoma, hepatocellular carcinoma, cholangiocarcinoma, gallbladder cancer and gastric cancer [24-30]. For examples, AFAP1-AS1 knockdown inhibited the nasopharyngeal carcinoma cell migration, invasive capability and AFAP1-AS1 promoted cancer cell metastasis via regulation of actin filament integrity [29]. In hepatocellular carcinoma, inhibited expression of AFAP1-AS1 induced cell apoptosis and blocked cell cycle in S phase via inhibition of the RhoA/Rac2 signaling [30]. Based on these previous findings, AFAP1-AS1 maybe has the potential to serve as a useful and promising diagnosis tool and therapy target for cancers. However, little is known about the regulatory role of AFAP1-AS1 in lung cancer. Additional investigations on AFAP1-AS1 would be performed to further disclose and support its potential as a novel noncoding RNA (ncRNA) biomarker in cancer clinical diagnosis and targeted therapy.

In this study, we investigated the expression level of lncRNA AFAP1-AS1 as well as its association with lung cancer progression. Our results revealed that AFAP1-AS1 was substantially over expressed in lung cancer tissues compared with that in adjacent normal tissues. In addition, AFAP1-AS1 expression was proved to be associated with histology type, tumor size, lymph node metastasis, distant metastasis and TNM stage. The results of knockdown experiments showed that knockdown of AFAP1-AS1 could inhibit cell proliferation and migration in lung cancer cells in *vitro* and suppress lung tumor growth in *vivo*. Moreover, we found that down-regulation of AFAP1-AS1 could influence the expression of AFAP1 and KRT1 on mRNA and protein level by *cis* and *trans* regulatory mechanism. The demonstration of the oncogenic function in lung cancer of lncRNA AFAP1-AS1 in our present study provided a valuable resource for understanding the role of AFAP1-AS1 in the development and progression of cancer.

## RESULTS

### AFAP1-AS1 expression is upregulated in lung cancer tissues and cell lines

**Figure 1:**
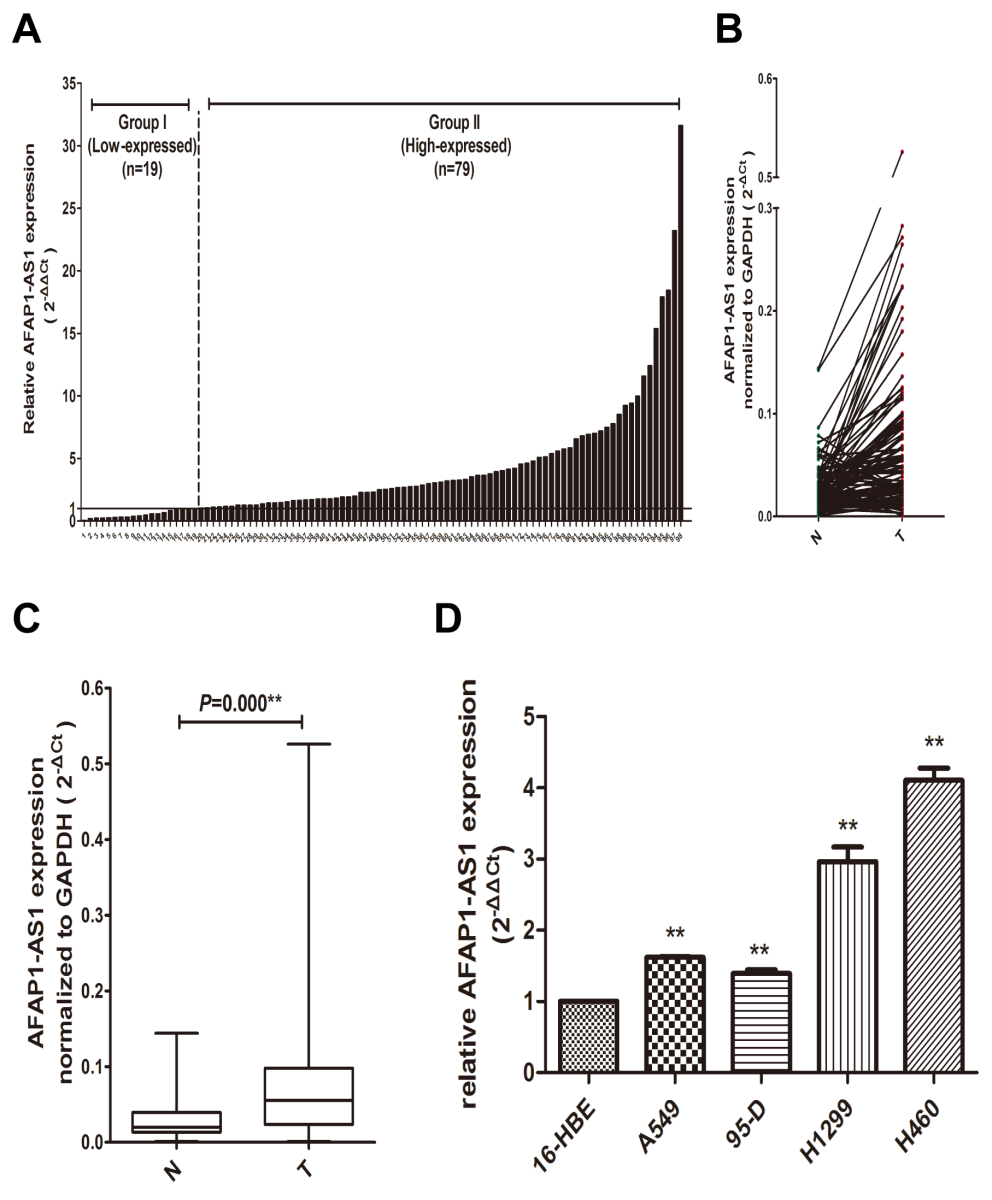
Overexpression of AFAP1-AS1 in lung cancer tissues and cell lines **(A)** AFAP1-AS1 expression in 98 pairs lung cancer and adjacent non-tumor lung tissues using qRT-PCR. **(B)** The comparison of AFAP1-AS1 expression between matched lung cancer and adjacent non-tumor lung tissues in 98 patients.**(C)** The data for AFAP1-AS1 expression were analyzed using the Mann-Whitney U-test. **(D)** The AFAP1-AS1 expression in lung cancer cell lines was determined via qRT-PCR, and GAPDH was used as internal control. Data are represented as the mean ± s.d. from three independent experiments. ^*^: *p* < 0.05; ^**^: *p* < 0.01. *P* values were obtained by one-way ANOVA or the non-parametric Kruskal-Wallis *H* test for multiple comparisons.

To identify the role of AFAP1-AS1 in lung cancer, we first examine the expression level of AFAP1-AS1 in 98 paired lung cancer samples and adjacent normal tissues using by qRT-PCR. The results indicated that the expression level of AFAP1-AS1 was significantly upregulated in cancerous tissue of 80.61% (79/98) lung cancer patients comparing with paired non-tumor tissues (*p* < 0.05; Figure 1A and 1B), with a median difference of approximately 2.44-fold (*p* < 0.05; Figure 1C). To confirm the association between the expression of AFAP1-AS1 and lung cancer, we also examined the expression of AFAP1-AS1 in multiple lung cancer cell lines, including A549, 95-D, H1299 and H460. As shown in Figure 1D, qRT-PCR results showed that the expression of AFAP1-AS1 was also significantly upregulated in A549 (1.54-fold, *p* < 0.05), 95-D (1.47-fold, *p* < 0.05), H1299 (2.79-fold, *p* < 0.05) and H460 (4.06-fold, *p* < 0.05) cells compared with normal human bronchial epithelial cell line 16HBE. This result was consistent with the findings obtained from lung cancer tissues.

We next examined the relationship between AFAP1-AS1 expression and the clinicopathological characteristics of the tumor tissue samples. As shown in Figure 1A, 98 lung cancer patients were separated into high-expressed group (fold change ≥1.0, n = 79) and low-expressed group (fold change < 1.0, n = 19) in our relationship analysis. Clinicopathologic features of 98 lung cancer patients and its detailed relationships with AFAP1-AS1 expression level are given in Table 1. As a result, AFAP1-AS1 high-expressed group showed greater histology type (*p* = 0.02), tumor size (*p* = 0.013), lymph node metastasis (*p* = 0.022), distant metastasis (*p* = 0.013), and TNM stage (*p* = 0.007) compared with the low-expressed group. However, AFAP1-AS1 expression level was not associated with other parameters, such as gender (*p* = 0.746), age (*p* = 0.711), smoking (*p* = 0.132) and histological cell type (*p* = 0.063). These results suggested that the over expression of AFAP1-AS1 is clinically relevant to the progress of lung cancer and maybe act as an oncogene in lung cancer.

**Table 1:** The relationship between lncRNA AFAP1-AS1 expression and the clinicopathological factors in 98 lung cancer patients

Clinicopathologic factor	Group I	Group II	*P*-value
	(<1.0)	(≥1.0)	
	(n=19)	(n=79)	
Gender			
Male	12	53	0.746
Female	7	26	
Age			
≥60	10	38	0.711
<60	9	41	
Smoking			
Yes	6	40	0.132
No	13	39	
Histologic cell type			
Well differentiated	2	31	0.063
Moderately differentiated	7	19	
Poorly differentiated	10	29	
Histology type			
Adenocarcinoma	4	45	0.020
Squamous carcinoma	12	26	
Small cell lung cancer	3	8	
Tumor size			
≥3	5	46	0.013
<3	14	33	
Lymph node metastasis			
Absent	12	27	0.022
Present	7	52	
Distant metastasis			
Absent	14	32	0.013
Present	5	47	
TNM stage			
I	13	22	0.007
II/III/IV	6	57	

### Down-regulation of AFAP1-AS1 by siRNA transfection

To investigate the functional involvement of AFAP1-AS1 in lung cancer, AFAP1-AS1 was silenced in H1299 cell line by siRNAs. We designed four targeting short interfering RNAs (siRNA-1, siRNA-2, siRNA-3 and siRNA-4) for knockdown experiment. To obtain the best interference efficiency for the next functional experiments, H1299 cells were then transfected with these four siRNAs by different concentrations (30 nM, 50 nM, 80 nM and 100 nM), respectively. Transfected cells were collected and the efficiency of interference was determined by qRT-PCR at the time point of 24 h and 48 h after transfection.

**Figure 2:**
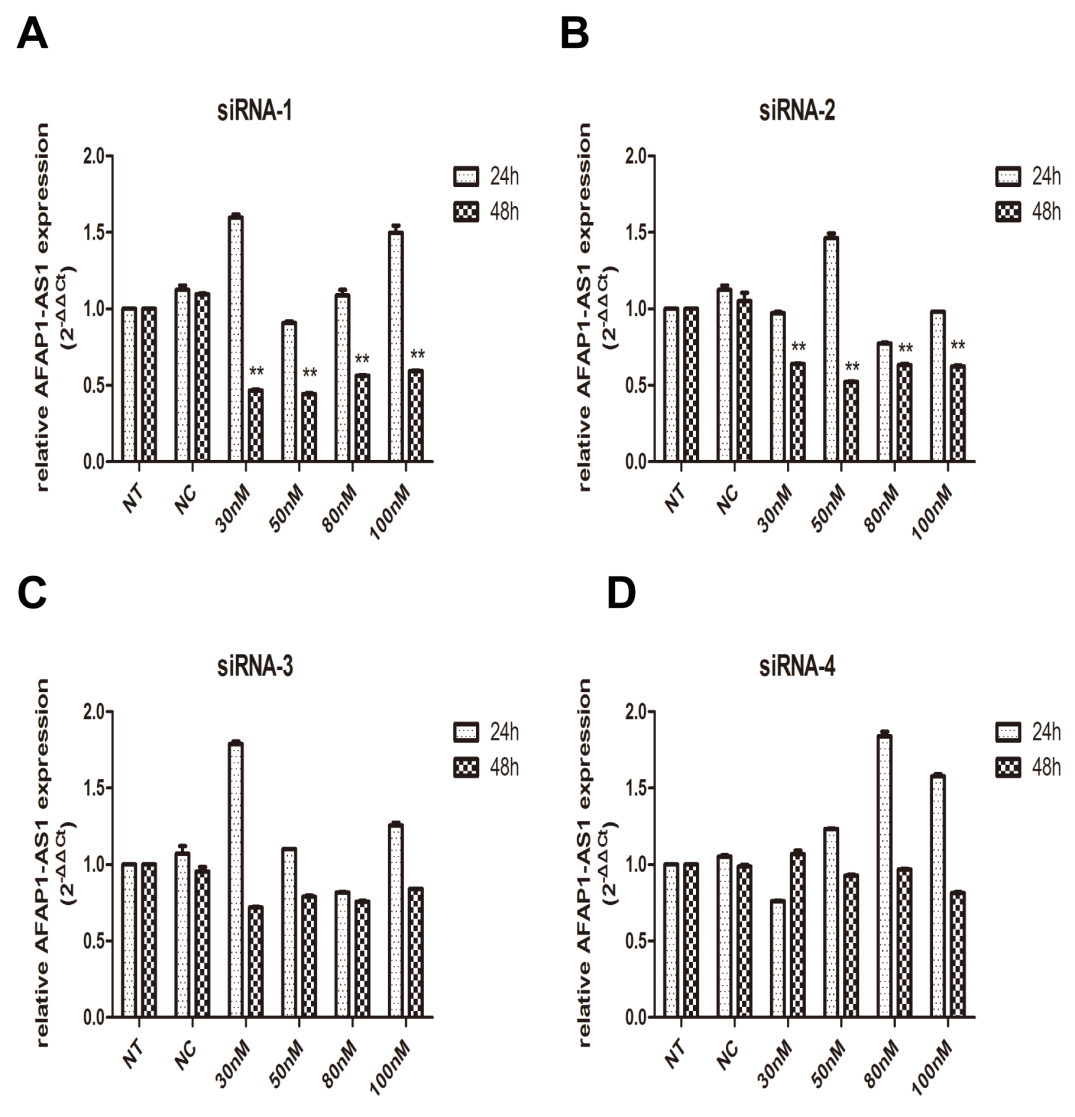
Knockdown of AFAP1-AS1 by siRNA transfection **(A)-(D)** Knockdown efficiency of AFAP1-AS1 siRNA-1(A), siRNA-2(B), siRNA-3(C) and siRNA-4(D) transfection was determined at RNA level by qRT-PCR in H1299 cells, compared with siRNA-NC and NT group. Data are represented as the mean ± s.d. from three independent experiments. ^*^: *p* < 0.05; ^**^: *p* < 0.01. *P* values were obtained by one-way ANOVA or the non-parametric Kruskal-Wallis *H* test for multiple comparisons.

We first examined the efficiency of four siRNA sequences in silencing AFAP1-AS1 24 h after transfection, all these four siRNAs showed merely a limited effect of silencing activities compared to siRNA-NC. Therefore, we further examined the effects of these RNAi components on AFAP1-AS1 silencing 48 h after transfection. As expected, significant improvement of silencing efficacy was observed. H1299 cells showed 56% and 58% inhibition of AFAP1-AS1 after transfected with 50 nM siRNA-1 and siRNA-2, respectively (Figure 2A and 2B). However, as shown in Figure 2C and 2D, we didn’t observe the similar silencing efficiency in siRNA-3 and siRNA-4 transfected cells. Therefore, a concentration of 50 nM for siRNA-1and siRNA-2 transfection was used in the subsequent experiments.

### AFAP1-AS1 knockdown inhibits cell growth, migration in *vitro*

**Figure 3:**
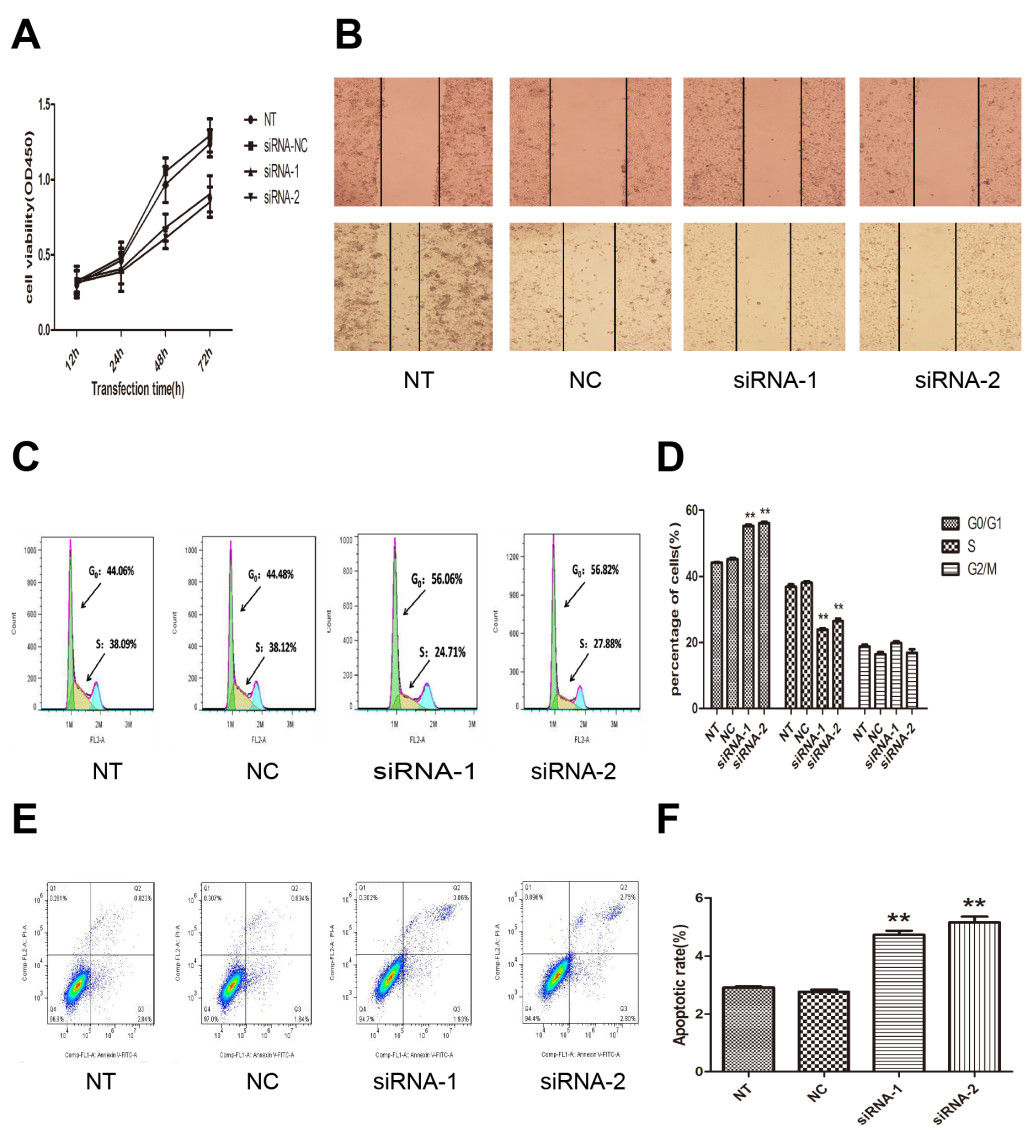
Effect of AFAP1-AS1 knockdown on cell proliferation, migration, cell cycle distribution and apoptosis in *vitro* **(A)** AFAP1-AS1 knockdown inhibited cell proliferation determined by CCK-8 assays in H1299 cells. **(B)** Representative photographs of wound healing assay were taken at 0 h and 24 h after the wound was made in the NT, siRNA-NC, siRNA-1, siRNA-2 transfected H1299 cells. **(C)-(D)** The effect of AFAP1-AS1 knockdown on cell cycle distribution was measured by flow cytometry. **(E)-(F)** The effect of AFAP1-AS1 knockdown on cell apoptosis was measured by flow cytometry. Data are represented as the mean ± s.d. from three independent experiments. ^*^: *p* < 0.05; ^**^: *p* < 0.01. *P* values were obtained by one-way ANOVA or the non-parametric Kruskal-Wallis *H* test for multiple comparisons.

To examine the functional role of AFAP1-AS1 in lung cancer progression, CCK-8 assay was first used to detect the impact of AFAP1-AS1 knockdown on proliferation in H1299 cells. As shown in Figure 3A, the cell growth curves of siRNA-1 and siRNA-2 group were all dramatically suppressed from 48 h transfected, compared with siRNA-NC group or NT group. The cell growth curve indicated that AFAP1-AS1 siRNA-transfected cells showed a significantly slower proliferation rate (*p* < 0.05). Consistent with proliferation observations, wound healing assays demonstrated that the migratory potential of H1299 cells following AFAP1-AS1 knockdown was significantly inhibited when compared with siRNA-NC group and NT group (*p* < 0.05; Figure 3B).

Next, flow cytometric analysis was performed to further address whether the deceleration of proliferation by AFAP1-AS1 knockdown might be a result of cell cycle arrest or cell apoptosis inducing. The cell-cycle progression results revealed that AFAP1-AS1 knockdown induced an accumulation of H1299 cells in the percentage of cells at the G0/G1 phase (siRNA-1 group, 56.06%; siRNA-2 group, 56.82%) compared with cells transfected with siRNA-NC (44.06%) (*p* < 0.05; Figure 3C and 3D). The results showed that the apoptosis rate of H1299 cells were increased in siRNA-1 group (5.16 ± 0.34%), siRNA-2 group (4.74 ± 0.24%) compared siRNA-NC group (2.77 ± 0.14%, *p* < 0.05; Figure 3E and 3F), or with NT group (2.9 ± 0.07%, *p* < 0.05; Figure 3E and 3F). These findings indicated that the AFAP1-AS1 knockdown induced cell cycle arrest and apoptosis in lung cancer cells, which contributes to demonstrate the growth promotion properties of AFAP1-AS1.

### AFAP1-AS1 knockdown inhibits tumor growth in *vivo*

**Figure 4:**
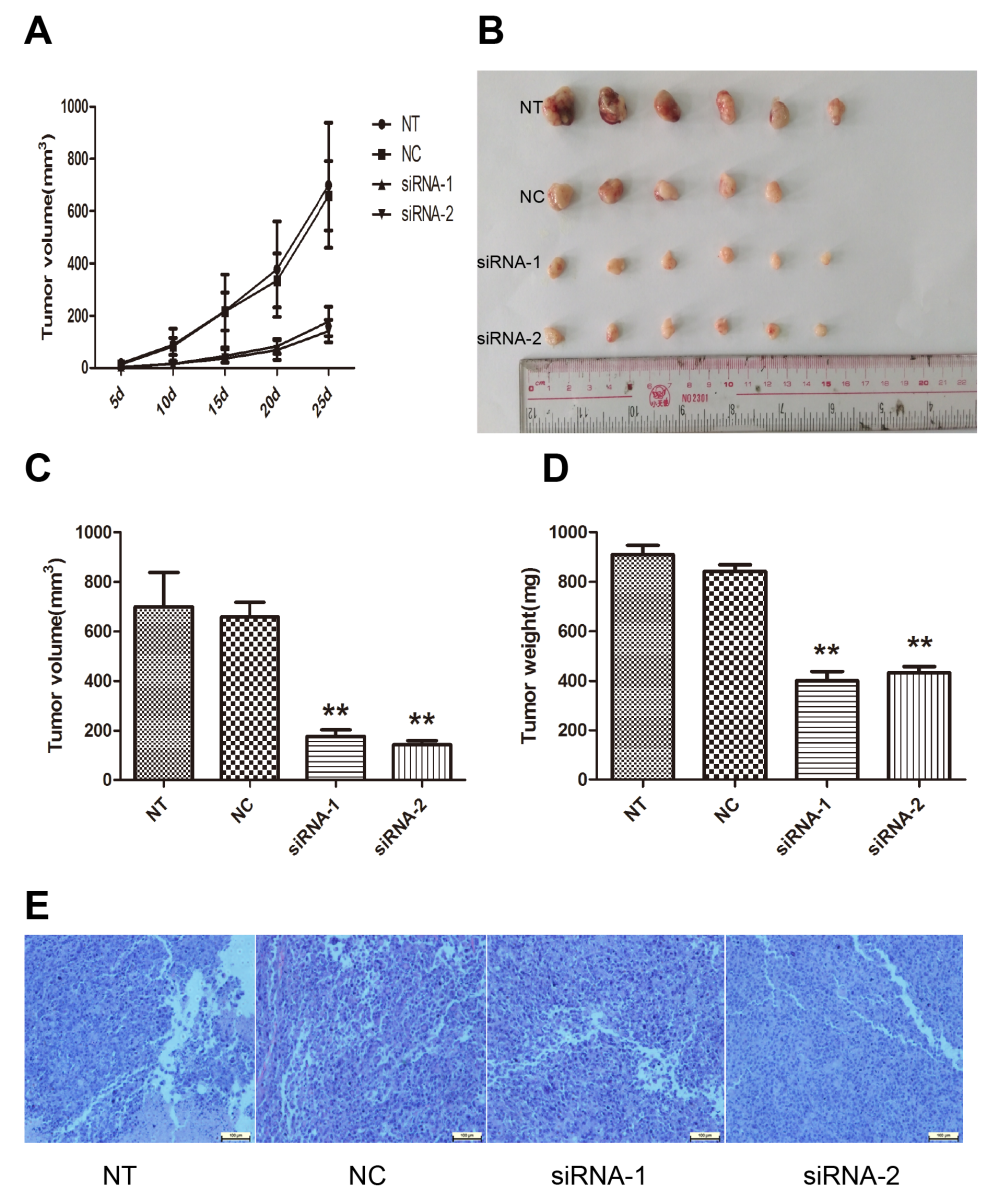
Effect of AFAP1-AS1 knockdown on tumor growth in *vivo* **(A)** Tumor growth curves were constructed after nude mice were injected with NT, si-NC and siRNA transfected H1299 cells. **(B)** Representative photograph of tumors derived from NT, si-NC and siRNA of AFAP1-AS1 groups. **(C)** Tumor volume was calculated with the formula V=0.5ab^2^, where “a” is the length of the tumor, “b” is the width of the tumor, and “V” is the mean tumor volume. **(D)** The comparison of tumor weight between NT, si-NC and siRNA groups. **(E)** Pathological examination of tumors with hematoxylin and eosin staining. Data are represented as the mean ± s.d. from three independent experiments. ^*^: *p* < 0.05; ^**^: *p* < 0.01. *P* values were obtained by one-way ANOVA or the non-parametric Kruskal-Wallis *H* test for multiple comparisons.

To further evaluate the role of AFAP1-AS1 in the lung cancer tumorigenesis, AFAP1-AS1 knockdown cells were subcutaneously injected into BALB/c nude mice as described in the materials and methods section. Xenograft tumors appeared at the injection site at eight days after injection in each group, including siRNA-1, siRNA-2, siRNA-NC and NT groups. Then, tumor growth was measured every five days and we found that tumors derived from cells transfected with the AFAP1-AS1 siRNA grew substantially more slowly than siRNA-NC group throughout tumor growth (Figure 4A). When the tumors were harvested 25 days after injection, it is of considerable interest that tumor volume of siRNA-1 group (178.27 ± 56.35mm^3^, *p* < 0.05, Figure 4B and 4C) and siRNA-2 group (141.11 ± 43.02 mm^3^, *p* < 0.05, Figure 4B and 4C) was significantly smaller than that of the si-NC group (658.88 ± 132.65 mm^3^). Consistently, the average weight of tumor of siRNA-1 group (400.75 ± 91.49 mg, *p* < 0.05; Figure 4D) and siRNA-2 group (433.04 ± 60.01mg, *p* < 0.05; Figure 4D) were less than that of si-NC group (908.93 ± 93.06 mg). There was no significant difference of tumor volume, tumor average weight or other indices between the siRNA-NC transfected group and the untransfected (NT) group. Additionally, pathological examination revealed that the mice injected with the four groups of cells developed adenocarcinoma cell carcinomas (Figure 4E). Taken together, our data suggested that AFAP1-AS1 knockdown could suppress tumor growth of lung cancer in *vivo*.

### Regulating role of AFAP1-AS1 on AFAP1 expression in *cis*

**Figure 5:**
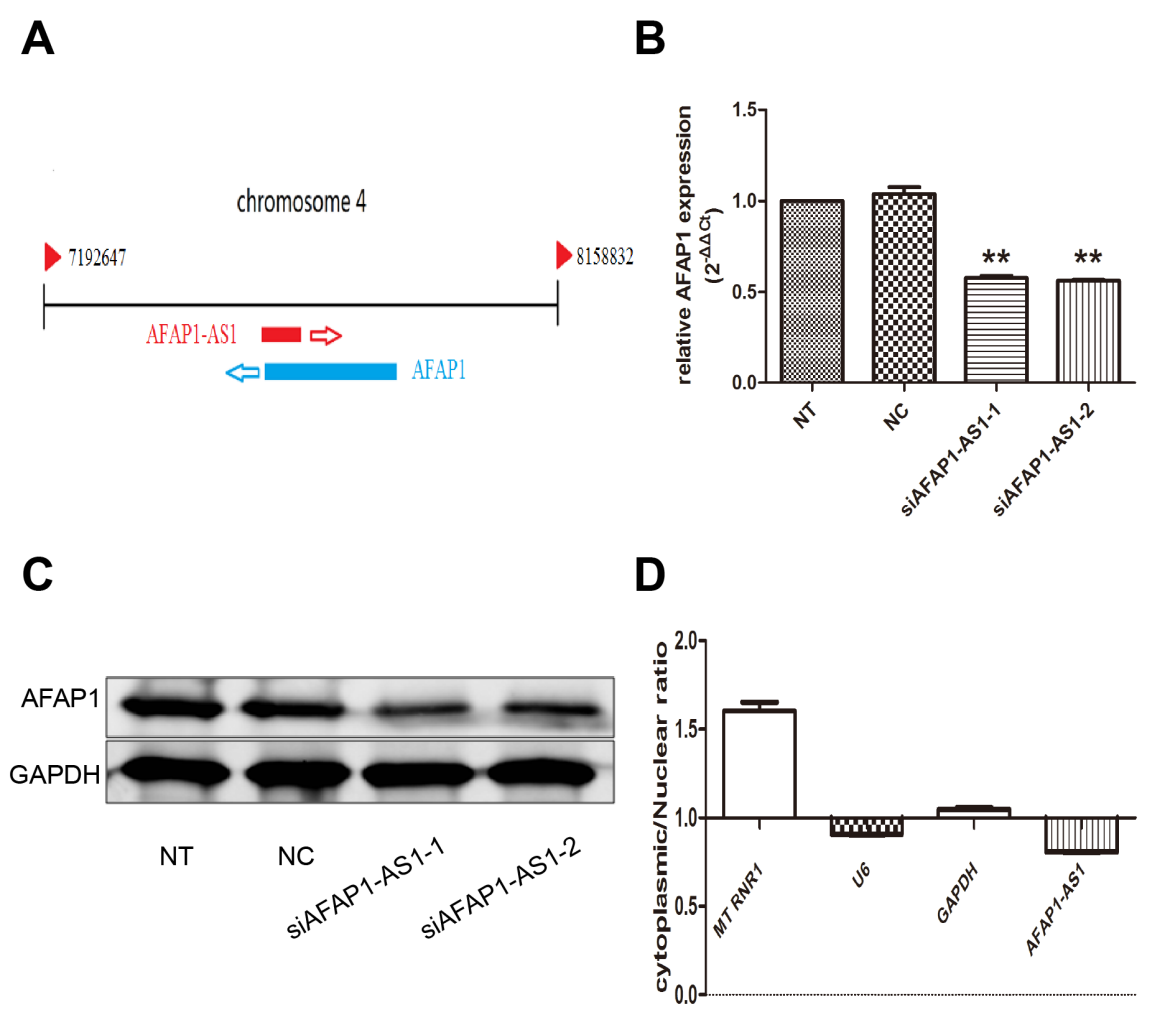
Regulating role of AFAP1-AS1 on AFAP1 expression **(A)** Alignment of AFAP1-AS1 with the protein-coding AFAP1 gene. **(B)** AFAP1-AS1 knockdown suppressed AFAP1 RNA levels in H1299 cells. **(C)** AFAP1-AS1 knockdown suppressed AFAP1 protein levels in H1299 cells. **(D)** Relative expression of AFAP1-AS1 in the nuclear and cytoplasmic fractions of H1299 cells was determined by qRT-PCR. Data are represented as the mean ± s.d. from three independent experiments. ^*^: *p* < 0.05; ^**^: *p* < 0.01. *P* values were obtained by one-way ANOVA or the non-parametric Kruskal-Wallis *H* test for multiple comparisons.

As shown in Figure 5A, AFAP1-AS1 localized at the antisense DNA strand of the gene coding AFAP1 protein and there are overlapping regions between the second AFAP1-AS1 exon and AFAP1 exon 14, 15 and 16. Thus, we speculated that AFAP1-AS1 might influence the expression of protein coding gene AFAP1. To address this assumption, expression of AFAP1 both on mRNA and protein levels were detected by qRT-PCR and western blotting after transfection with siRNA-1 and siRNA-2 in H1299 cells. Our results revealed that AFAP1-AS1 knockdown in H1299 cells significantly down regulated the expression of AFAP1 in siRNA-1 group (*p* < 0.05) and siRNA-2 group (*p* < 0.05), compared with siRNA-NC or NT group (Figure 5B). Meanwhile, down regulation of AFAP1 was further confirmed by western blotting in protein level. We found that the protein expression level of AFAP1 was also obviously suppressed in siRNA-1 group and siRNA-2 group compared with siRNA-NC and NT groups (Figure 5C). Moreover, the result of intracellular localization assay indicated that AFAP1-AS1 was predominantly in the nucleus, because that the AFAP1-AS1 content in the cell nucleus was at least twice that in the cytoplasm (*p* < 0.05; Figure 5D).These findings implied that AFAP1-AS1,localized primarily in the nucleus, might mainly affect AFAP1 expression at transcriptional level by *cis*-regulatory mechanism and resulted in AFAP1 protein change accordingly.

### Regulating role of AFAP1-AS1 on KRT1 expression in *trans*

**Figure 6:**
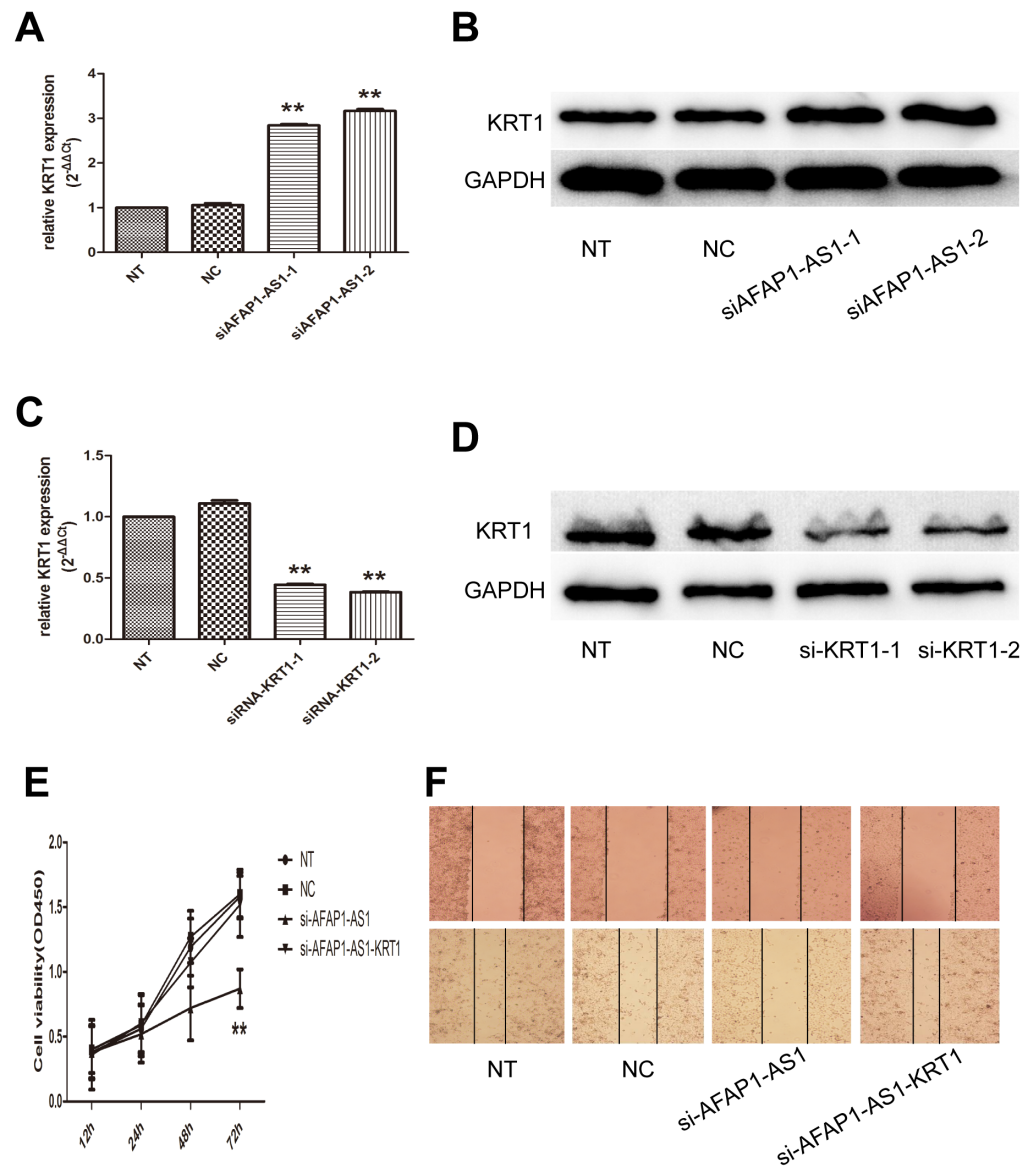
Regulating role of AFAP1-AS1 on KRT1 expression **(A)** AFAP1-AS1 knockdown upregulated KRT1 RNA levels in H1299 cells. **(B)** AFAP1-AS1 knockdown upregulated KRT1 protein levels in H1299 cells. **(C)** Si-KRT1 down regulated KRT1 expression after AFAP1-AS1 knockdown at the RNA level when compared with siRNA-NC group in H1299 cells by qRT-PCR. **(D)** Si-KRT1 downregulated KRT1 expression after AFAP1-AS1 knockdown at the protein level. **(E)** Cell viability curve determined in H1299 cells co-transfected with siRNA-KRT1 and siRNA-AFAP1-AS1 by CCK-8 assays. **(F)** Representative photographs of wound healing assay were taken at 0 h and 24 h after the wound was made in the NT, NC, si-AFAP1-AS1 and si-AFAP1-AS1-KRT1 transfected H1299 cells. Data are represented as the mean ± s.d. from three independent experiments. ^*^: *p* < 0.05; ^**^: *p* < 0.01. *P* values were obtained by one-way ANOVA or the non-parametric Kruskal-Wallis *H* test for multiple comparisons.

To explore the regulatory potential in *trans* of AFAP1-AS1 in lung cancer, we performed RNA antisense purification and mass spectrometry (RAP-MS) to characterize the proteins that bind directly to AFAP1-AS1 in *vivo*. Bound proteins were liberated from the RNA by treatment with RNase and identified by mass spectrometry. Lysates from negative control-infected cells were processed in parallel to aid in the elimination of nonspecific background during the evaluation of mass spectrometry data. Data for all samples from three independent experiments, including the numbers of unique peptides for each of 132 proteins identified, are shown in Supplementary Table 1 in the supplemental material. Based on the Mascot Score Histogram and protein score, we derived a list of 20 candidate proteins that may be associated with AFAP1-AS1 *in vivo* (Table 2). KRT1, which has the highest protein score (score = 218) in our RAP-MS analysis, was selected to validate the interaction effect. As expected, we observed that AFAP1-AS1 knockdown resulted in a significantly increase of KRT1 mRNA (*p* < 0.05; Figure 6A) and protein (Figure 6B).

**Table 2:** List of 20 candidate proteins that may be associated with lncRNA AFAP1-AS1

Protein access	Protein description	Protein score
K2C1_HUMAN	Keratin, type II cytoskeletal 1	218
K22E_HUMAN	Keratin, type II cytoskeletal 2	214
EFA1A_HUMAN	Elongation factor 1-alpha 1	101
MYH9_HUMAN	Myosin-9	96
K2C5_HUMAN	Keratin, type II cytoskeletal 5	96
DMBT1_HUMAN	Deleted in malignant brain tumors	95
TRFL_HUMAN	Lactotransferrin	82
K1C10_HUMAN	Keratin, type I cytoskeletal 10	75
TBA1C_HUMAN	Tubulin alpha-1C chain	73
TBA1A_HUMAN	Tubulin alpha-1A chain	73
K2C6C_HUMAN	Keratin, type II cytoskeletal 6c	72
K1C17_HUMAN	Keratin, type I cytoskeletal 17	71
K2C6B_HUMAN	Keratin, type II cytoskeletal 6b	71
FIBG_HUMAN	Fibrinogen gamma chain	69
K2C3_HUMAN	Keratin, type II cytoskeletal 3	67
K1C9_HUMAN	Keratin, type I cytoskeletal 9	63
ROA2_HUMAN	Heterogeneous nuclear ribonucleopreteins	61
K220_HUMAN	Keratin, type II cytoskeletal 2	58
K2C79_HUMAN	Keratin, type II cytoskeletal 79	58
FINC_HUMAN	Fibronectin	56

To test whether KRT1 found to bind AFAP1-AS1 play a role in mediating regulatory function of AFAP1-AS1, we performed siRNA-mediated gene silencing followed by AFAP1-AS1 knockdown. The efficacy of KRT1 silencing in AFAP1-AS1 knockdown cells was confirmed at both the mRNA (*p* < 0.05; Figure 6C) and protein levels (Figure 6D). We found that AFAP1-AS1 and KRT1 knockdown was associated with a significant increase in cell numbers assessed with a CCK-8 assay, compared to AFAP1-AS1 knockdown cells (*p* < 0.05; Figure 6E). Furthermore, KRT1 siRNA enhance the migratory potential of H1299 cells, which was decreased upon the transfection of AFAP1-AS1 siRNA (Figure 6F). This is the evidence that the oncogenic activities of AFAP1-AS1 are mediated, at least in part, by directly limiting KRT1 expression.

## DISCUSSION

To date, lung cancer is the leading cause of cancer-related deaths worldwide in both men and women. Recent advances over the understanding of molecular mechanisms involved in pathogenesis of lung cancer are leading to rapid development of clinical treatment. Despite this, the prognosis of lung cancer patients with advanced stage (IIIB and IV) remains unfavorable, with a 5-year worldwide survival rate of 5% and 1 %, respectively [31, 32]. The limited availability of biomarker is certainly main determinants of this poor clinical outcome. Extensive studies are needed to find new molecular biomarkers and therapeutic targets.

In our present study, lncRNA AFAP1-AS1 was found to actively be involved in lung cancer development and progression. Lung cancer patients with high AFAP1-AS1 expression levels presented with lymph node metastasis and advanced stage of disease, suggesting the relevance of AFAP1-AS1 up regulation in promoting lung cancer progression. Our results corroborate those of Jinyan and colleagues who showed that high AFAP1-AS1 expression levels in hepatocellular carcinoma (HCC) was associated with pathological staging, lymph-vascular space invasion, and a shorter median survival time [30]. Moreover, high AFAP1-AS1 level has also been identified as a negative prognostic factor, as it correlates with metastasis development and poorer overall survival in pancreatic ductal adenocarcinoma patients with surgical resection [25]. These results indicated that AFAP1-AS1 might function as an oncogene and exhibit important role development and progression of lung cancer. It may be useful as a novel prognostic marker for lung cancer.

In addition to the potential importance of AFAP1-AS1 as a marker of cancers, it may be a potential therapeutic target for cancer progression. AFAP1-AS1 inhibition by siRNA diminished cell proliferation and migration by increasing apoptosis and resulting in G0/G1-phase arrest in H1299 cell. Moreover, AFAP1-AS1 knockdown markedly reduced tumor growth and formed smaller tumor in xenograft mouse model. The dramatic pro-tumoral effect of AFAP1-AS1 demonstrated in our present study, either in culture or in xenografted nude mice, consistent with the results of some previous research on AFAP1-AS1. Early studies showed markedly upregulated expression levels of AFAP1-AS1 in Barrett’s esophagus and esophageal adenocarcinoma, with AFAP1-AS1 silencing by small interfering RNA leading to inhibited proliferation and colony-forming ability, induced apoptosis, and reduced cell migration and invasion [24]. Recently, further investigation of some other AS lncRNAs may also contribute to our understanding of the role of AS lncRNAs in cancer biology. For example, DSCAM-AS1 was demonstrated to mediate tumor progression and tamoxifen resistance in breast cancer. Additionally, hnRNPL was identified as an interacting protein involved in the mechanism of DSCAM-AS1 action [33]. These data have been confirmed and expanded upon by several other studies [29, 30, 34-36], supporting the evidence that AFAP1-AS1 oncogenic activity could play a critical role in many types of cancer. Small interfering RNAs (siRNAs) that target RNA molecules via complementary to paired lncRNA sequences exhibited high knockdown effectiveness to lncRNA AFAP1-AS1 in lung cancer cells and reported encouraging anticancer effects both in *vitro* and in *vivo* in our study. Taken together, AFAP1-AS1 should been evaluated as potential therapeutic targets.

Recently, lncRNAs have gained widely attention as potentially crucial regulators of gene expression and new players in cancer biology [37, 38]. These lncRNAs contribute to cancer hallmark characteristics and operate transcriptionally, post transcriptionally, or epigenetically to regulate gene expression [39]. Through regulating gene expression by different mechanisms, including gene transcription, genomic imprinting and chromatin modification, lncRNAs play crucial roles in the regulation of multiple biological processes [40]. Our data demonstrated that lncRNA AFAP1-AS1 functions in both *cis* and *trans* mechanisms, regulating gene expression of genomically neighboring protein-coding genes in *cis* or of distant protein-coding genes in *trans*. AFAP1-AS1 is oriented in antisense direction with respect to a protein coding gene, AFAP1. We found that there was a positive correlation between AFAP1-AS1 and AFAP1 sense expression in lung cancer tissues and cell lines. Our present results implied that AFAP1 mRNA expression maybe under the control of lncRNA AFAP1-AS1. Antisense lncRNAs are usually reported to act as regulator of gene of the opposite strand [41, 42], but Wu et al. demonstrated that AFAP1-AS1 had limited effect on AFAP1 expression, instead functioning in an AFAP1-independent manner in Barrett’s esophagus and esophageal adenocarcinoma [24]. Further studies are needed to investigate whether AFAP1-AS1 is involved in regulating AFAP1 in different cancers*.*

To identify whether AFAP1-AS1 also modulate gene expression in *trans*, we used RAP-MS assays to purify AFAP1-AS1 complex and identify the majority of known RNA interacting proteins. As a result, we confirmed that AFAP1-AS1 specifically binds to Keratin, type II cytoskeletal 1 protein (KRT1) and loss of AFAP1-AS1 expression accelerated the expression of KRT1. KRT1 protein has been reported to play a tumor suppressive role in various cellular processes like proliferation, cell death, cell cycle, and cytoskeleton organization [43-46]. Hence, it can be assumed that the inhibited effect in cancer development induced by AFAP1-AS1 knockdown might be at least partly through the upregulated KRT1 in lung cancer. Although lncRNA AFAP1-AS1 is over expressed in various types of cancer, the target genes are rather variable and depend on the host cell type. Recently, AFAP1- AS1 has been demonstrated to regulate Rho/Rac GTPase family members and actin cytokeratin signaling pathway in lung cancer, which suggested that AFAP1-AS1 might affect tumor cell invasion and migration through the small GTPase pathway and the related kinase [47]. Recently, AFAP1-AS1 was reported to play oncogenic roles in gastric cancer by regulating cell proliferation and apoptosis via PTEN/p-AKT pathway [48]. To date, the regulation of AFAP1-AS1 and its involvement in tumor progression has not widely been disclosed. LncRNAs have been shown to be functional through their binding interactions with other DNA, RNA and proteins. Thus, identifying more binding partners for AFAP1-AS1 is a crucial step in understanding the oncogenic role of lncRNA AFAP1-AS1 in the future study.

In conclusion, we demonstrated the novel oncogenic lncRNA AFAP1-AS1 played a crucial role in lung cancer progression and development either in culture or in xenografted nude mice. We also provided evidence that AFAP1-AS1 regulated the expression of AFAP1 in *cis* and may interact with KRT1 in *trans*. Although lncRNA AFAP1-AS1 represented a potentially promising biomarker and provided a new therapeutic tool in treating malignant tumors, more efforts to explore the mechanism of its action in cancer pathogenesis were needed in future research.

## MATERIALS AND METHODS

### Tissue samples and clinical data collection

98 pairs of lung cancer and adjacent non-tumor lung tissues were obtained from patients who received surgical resection between 2013 and 2015 at Department of Thoracic Surgery, General Hospital of Guangzhou Military Command of Chinese People’s Liberation Army (Guangzhou, China). The non-tumor lung tissues were sampled at a distance of 3 cm away from the tumor. Tissues were fresh-frozen in liquid nitrogen immediately after resection and then stored at −80°C before RNA extraction. None of these patients received any preoperative chemotherapy or radiotherapy. All clinicopathological data were obtained from patient clinical and pathologic records. The use of tissues for this study has been approved by the ethics committee of General Hospital of Guangzhou Military Command of Chinese People’s Liberation Army and Guangzhou Medical University. Before using these clinical materials of research purposes, all the patients signed the informed consent.

### Cell culture

Three lung cancer cell lines (A549, H1299 and H460) were purchased from the American Type Cell Culture (Rockville, MD, USA). Lung cancer cell lines 95-D and the human bronchial epithelial cell line 16HBE were kindly provided by the Guangzhou Institute of Respiratory Disease (Guangzhou, China). Cells were cultured in RPMI 1640 (Invitrogen, Carsbad, CA, USA) supplemented with 10% fetal bovine serum and 1% antibiotics (penicillin/streptomycin) in humidified air at 37°C with 5% CO_2_. All the cell lines used in this study tested positive for human origin using short tandem repeat (STR) markers, morphology check, growth curve analysis, and chromosome karyotyping by Genetic Testing Biotechnology Corporation (Suzhou, China) in April, 2016 before use for this study.

### RNA extraction and qRT-PCR analyses

Total RNA was extracted from tissues or cultured cells using TRIzol Reagent (Invitrogen, Carlsbad, CA) according to the manufacturer’s protocol. RNA quality and concentration were determined by measuring the absorbance at 260 nm (A_260_) and 280 nm (A_280_) with a Nanodrop ND-1000 spectrophotometer (Nanodrop Technologies, Montchanin, DE, USA). For qRT-PCR, RNA was reverse transcribed to cDNA by using a GoScript Reverse Transcription System (Promega, Madison, USA). Real-time PCR analyses were performed with GoTaq qPCR Master Mix (Promega, Madison, USA). The qRT-PCR cycling conditions were as follows: polymerase activation at 95°C for 2 min, 40 cycles at 95°C for 15 s, and 60°C for 1 min. PCR products were identified by melting curve analysis. The data were calculated using the 2^−ΔΔCt^ method. Results were normalized to the expression of GAPDH. All primers were synthesized by Invitrogen (Shanghai, China). The primer sequences for qRT-PCR are as follows:

AFAP1-AS1 Forward 5′ - GGAGTGACGGCATCCAACTC - 3′,
     Reverse 5′ - GTCATCCCTGTCCCTGGTTC;
AFAP1 Forward 5′- CCGTGCATCAACGGCTCGCTC -3′,
     Reverse 5′ - TTCACAACAGCCGCGGGATCC - 3′;
KRT1 Forward 5′ - GATTGCCACCTACAGGACCC - 3′,
     Reverse 5′ – ACAGACACACTCACGTTCGG – 3′;
GAPDH Forward 5′ - CAATGACCCCTTCATTGACC - 3′,
     Reverse 5′ - GACAAGCTTCCCGTTCTCAG - 3′;
U6 Forward 5′ - CCTCCCCAATAAAGCTAAAA - 3′,
     Reverse 5′- GCTATTGTGTGTTCAGATAT - 3′;
MT RNR1 Forward 5′ - TGCTCGCTTCGGCAGCACAT - 3′,
     Reverse 5′- CTTGCGCAGGGGCCATGCTA - 3′.

### RNA interference

Four pairs of small interfering RNAs (siRNAs) were designed to knockdown the expression of AFAP1-AS1, designated siRNA-1, siRNA-2, siRNA-3, and siRNA-4 and one pair of scrambled siRNAs (siRNA-NC), and three siRNAs were designed to knockdown KRT1 expression, designated si-KRT1-1, si-KRT1-2 and si-KRT1-3. All siRNAs were synthesized by Ribobio (Guangzhou, China). H1299 cells were grown up to 60% confluence in 6-well plates and then transfected with siRNA or siRNA-NC at various concentrations (30 nM, 50 nM, 80 nM and 100nM) using Lipofectamine 3000 (Invitrogen, Carlsbad, CA, USA), according to the manufacturer’s instructions. The sequences of siRNA primers were as follows:

siRNA-1 5′ - GGGCUUCAAUUUACAAGCA - 3′,
    5′ - UGCUUGUAAAUUGAAGCCC- 3′;
siRNA-2 5′ - CUCGUUGUGAAACUUAAAU - 3′,
    5′ - AUUUAAGUUUCACAACGAG - 3′;
siRNA-3 5′ - GGUGGAGAAUGAACAUUCU - 3′,
    5′ - AGAAUGUUCAUUCUCCACC - 3′;
siRNA-4 5′ - GCCGAUGAAAGUGUCUGAU - 3′,
    5′ - AUCAGACACUUUCAUCGGC - 3′;
siRNA-NC 5′ - UUCUCCGAACGUGUCACGU - 3′,
    5′ - ACGUGACACGUUCGGAGAA - 3′;
si-KRT1-1 5′ - GCACAAATGCAGAGAATGA - 3′,
    5′ - UCAUUCUCUGCAUUUGUGC - 3′;
si-KRT1-2 5′ – GTAGATACCTCCACTAGAA - 3′,
    5′ - UUCUAGUGGAGGUAUCUAC - 3′;
si-KRT1-3 5′ - GGATAGTGTGAGAAATTCA - 3′,
    5′ - UGAAUUUCUCACACUAUCC - 3′.
Total RNA was prepared 24 h and 48 h after transfection and was used for qRT-PCR to confirm the knockdown efficiency of the siRNAs. Three replicates were performed per time point in each experiment.

### Cell proliferation assays

Transfected H1299 cells were seeded onto 96-well plates and cell viability was tested with Cell Counting Kit-8 (CCK-8; Dojindo, Tokyo, Japan) according to the manufacturer’s instruction. 10 μl of CCK-8 reagent was added to each well after incubation for 12 h, 24 h, 48 h and 72 h. After incubation for 1 h - 2 h, A_450_ was measured for each well using a Synergy 2 microplate reader (BioTek, Winooski, VT). Cell viability (% of control) was calculated as (OD_test_ – OD_blank_)/(OD_control_ – OD_blank_), where OD_test_ is the optical density of the transfected cells, OD_control_ is the optical density of the siRNA-NC-transfected cells or cells without treatment, and OD_blank_ is the optical density of the wells without cells. Each experiment was performed in 3 replicates and repeated at least thrice.

### Flow-cytometric analysis of apoptosis and the cell cycle

Annexin V-fluorescein isothiocyanate (FITC) assay was used to assess apoptosis by flow cytometry. H1299 cells transiently transfected with siRNA or siRNA-NC were harvested 48h after transfection by trypsinization. After the double staining with FITC-Annexin V and propidium iodide (PI), the cells were analyzed with a flow cytometry (FACScan; Becton Dickinson, Franklin, NJ). Cells were discriminated into viable cells, dead cells, early apoptotic cells and apoptotic cells. Then the relative ratio of early apoptotic cells was compared to control transfectant from each experiment. For the cell cycle, cells were washed 2 times with cold PBS and fixed in 70% (v/v) cold ethanol overnight. Cells for cell-cycle analysis were stained with propidium iodide (PI) and finally analyzed immediately with flow cytometry. Cell cycle phases were assigned to G0/G1, S and G2/M according to the amount of DNA. The data were gated with the FlowJo software (Tree Star Inc., Ashland, OR, USA).

### Wound healing assay

The transfected cells were seeded and grown to 90% confluence in 6-well culture plates. A 200μl pipet tip was used to create a scratch in the cell monolayer and rinsed with PBS to remove the cellular debris. The average extent of wound closure after wounding was observed every 6 h for one day under an inverted microscope equipped with a CCD camera (Olympus, Japan). Representative photographs were taken at 0 h and 24 h after the wound was made. The experiment was performed in triplicate.

### Western blot assay

The protein content in the lysates was determined using the BCA protein assay (Beyotime Biotechnology, Shanghai, China) as manufacturer’s instructions. Equal protein amounts (30µg) were resolved through SDS-PAGE. Cells protein lysates were separated by 10% SDS-polyacrylamide gel electrophoresis (SDS-PAGE) and transferred to 0.22 μm PVDF membranes (Millipore, Massachusetts, USA). The PVDF membranes were then blocked by 5% skim milk containing 0.1% Tween-20 at room temperature for 1 h and then incubated overnight at 4°C with specific antibodies. After washing with TBST the next day, the membranes were incubated with secondary antibodies labeled with IRDye 800CW conjugated anti-rabbit IgG (Li-Cor, Lincoln, NE) at room temperature for 1-2 h. Autoradiograms were quantified by the Odyssey infrared Imaging System (Li-Cor). GAPDH (Abcam, Cambridge, UK) antibody was used as control. Anti-AFAP1 (1:1000) and anti-KRT1 (1:1000) were purchased from Abcam (Cambridge, UK).

### Tumor xenograft model

Four-week-old Balb/c nude mice were purchased from the Medical Animal Experimental Center of Southern Medical University (Guangzhou, China). All experimental procedures involving animals were performed in accordance with the institutional guidelines and approved by the Animal Care and Use Committee of Guangzhou Medical University. For xenograft models, 24 of the BALB/c nude mice were separated into four groups and six in each group, 1×10^6^ cells suspended of H1299 transfected with siRNA-1, siRNA-2, siRNA-NC and non-treated group were injected subcutaneously in the right flank of BALB/c nude mice. Tumor volumes were examined every 5 days when the implantations were starting to grow bigger. After 21 days, these mice were sacrificed by cervical dislocation and tumors were measured and fixed with 4% paraformaldehyde solution for histological analysis. Tumor volume was calculated as follows: Volume = (a×b^2^)/2, where a meant the longest diameter and b meant the shortest diameter.

### Intracellular localization assay

The nuclear and cytoplasmic fractions were isolated from 10^2^ - 10^7^ H1299 cells using the PARIS kit (Ambion, Austin, Texas, USA). Briefly, the cells were harvested by trypsinization, washed with cold PBS, added cell fractionation buffer, resuspended cells, incubated on ice, and centrifuged at 4°C. The cytoplasmic fraction was carefully aspirated and collected, while the nuclear pellet was lysed with disruption buffer. Then, each lysate was mixed an equal volume of 2 × lysis/binding solution. RNA was isolated from separate lysates with the addition of 100% ethanol and mixed through a filter cartridge. Applying wash solution 1 and wash solution 2/3 to the filter cartridge to wash the lysates. The nuclear and cytoplasmic RNAs (500 ng) were then converted to cDNA and analyzed with qRT-PCR.

### RNA affinity purification-mass spectrometry (RAP-MS)

AFAP1-AS1 RNA sequence was sequentially covered by the designed three pairs-nucleotide oligo without overlapping. Then, all individual probes was mixed together to create a probe stock to cover the length of the AFAP1-AS1 RNA. H1299 cells were seeded and grown to 80-90% confluence before UV cross-linking (∼19×10^6^ cells per dish). After washing cells twice with PBS, H1299 cells were irradiated with 0.15 J cm^−2^ (∼1 min) at 254-nmUV light for cCL or at 365-nm UV light for 4SU-labeled cells (PAR-CL). The cells were lysed in lysis buffer and resuspended after centrifuged. Magnetic beads and 3ug probe were added per tube to the lasete and incubate for 1h at 4°C. After stringent washed, the mRNA-protein complexes were eluted with elution buffer. RNase buffer, RNase T1 and RNase A were used to digest RNA from the mRNA-protein complexes. Before mass spectrometry, the samples are buffer-exchanged, concentrated, reduced, alkylated and digested using Amicon Ultra centrifugal filters according to the FASP protocol [49, 50]. At the end of this protocol, samples were ready for LC-MS measurements using a nanoflow LC system (Proxeno EASy-nLC1000) coupled to a hybrid linearion trap Obritrap Elites mass spectrometer (Thermo Fisher Scientific) for the experiments.

### Statistical analysis

Statistical analysis was performed using SPSS version 16.0 (SPSS, Chicago, IL, USA), as well as GraphPad Prism Software (Graph Pad Software Inc., San Diego, CA, USA). Student’s t-test (two-tailed), one-way ANOVA and Mann-Whitney test were performed to analyze the *in vitro* and *in vivo* data. Values were stated in the format of means±SD. For all statistical analysis in this study, *p* values less than 0.05 were considered statistically significant.

## SUPPLEMENTARY MATERIALS TABLE




