# Supplementary material for: Silencing of lncRNA AFAP1-AS1 suppressed lung cancer development by regulatory mechanism in cis and trans
Source: Oncotarget. 2017 Aug 24;8(55):93608–23. doi: 10.18632/oncotarget.20549 (PMC5706822; doi:10.18632/oncotarget.20549)
Supplement: Supplementary file 1 [file oncotarget-08-93608-s001.pdf]

Silencing of lncRNA AFAP1-AS1 suppressed lung cancer development by regulatory mechanism in *cis* and *trans*

SUPPLEMENTARY MATERIALS

Supplementary Table 1: 132 proteins identified to bind with AFAP1-AS1 by RNA Affinity Purification-Mass Spectrometry.

See Supplementary File 1
